# Gut-Derived Metabolites and Cognitive Health: Roles of Short-Chain Fatty Acids and Trimethylamine N-oxide

**DOI:** 10.7759/cureus.103206

**Published:** 2026-02-08

**Authors:** Dharmendra Kumar Gupta, Rakesh Kumar

**Affiliations:** 1 Physiology, Burdwan Medical College, Burdwan, IND; 2 Community Medicine, IQ City Medical College and Hospital, Durgapur, IND

**Keywords:** alzheimer's disease, blood-brain barrier, cognitive decline, gut-brain axis, gut microbiota, mild cognitive impairment, neurodegeneration, neuroinflammation, short-chain fatty acids, trimethylamine n-oxide

## Abstract

The gut microbiota has emerged as an important regulator of host physiology, extending well beyond digestion and metabolism. Increasing attention has focused on the gut-brain axis, a bidirectional communication network linking the gastrointestinal tract and the central nervous system. Among the many microbial metabolites implicated in gut-brain signalling, short-chain fatty acids (SCFAs) and trimethylamine N-oxide (TMAO) have attracted particular interest because of their potential roles in neuroinflammation, vascular dysfunction, and cognitive decline.

This narrative review synthesizes current evidence linking SCFAs and TMAO to cognitive health, drawing on human observational studies, experimental animal models, and mechanistic and secondary syntheses. Human data remain limited and largely observational. Altered gut microbiota composition and reduced SCFA levels have been reported in Parkinson's disease and have been associated with disease severity and neurological phenotypes. In parallel, TMAO has been detected in human cerebrospinal fluid and shown to interact with the blood-cerebrospinal fluid barrier, establishing biological plausibility for central nervous system exposure. Observational studies further link circulating TMAO levels with Alzheimer's disease biomarkers, mild cognitive impairment, and dementia-related neuroimaging features.

Experimental evidence provides more direct support. TMAO supplementation promotes brain aging, cognitive impairment, and neuropathological changes in mouse and rat models. In contrast, SCFAs, particularly butyrate, exert neuroprotective effects in models of Alzheimer's disease, Parkinson's disease, and systemic inflammation, with improvements in memory and reductions in pathological markers.

Mechanistic studies suggest that SCFAs may modulate immune responses, preserve blood-brain barrier integrity, and regulate microglial activity, whereas TMAO has been linked to endothelial dysfunction, oxidative stress, and neurovascular impairment. Taken together, available evidence supports biologically plausible but still preliminary roles for gut-derived metabolites in cognitive health. SCFAs appear broadly neuroprotective, while TMAO shows adverse associations, particularly in preclinical models. Human causality remains unproven, and clinical translation is premature. Well-designed longitudinal and interventional studies are required before these metabolites can be considered reliable biomarkers or therapeutic targets.

## Introduction and background

The human gut microbiota plays a central role in host physiology. Beyond its well-established contributions to digestion and energy metabolism, it influences immune regulation, endocrine signalling, and neural function. Over the past decade, growing attention has focused on the gut-brain axis, a bidirectional communication network linking the gastrointestinal tract and the central nervous system. Disruption of this axis has been implicated in neurodegenerative disorders such as Alzheimer's disease and Parkinson's disease, as well as in age-related cognitive decline [1-3].

Among the molecular mediators involved in gut-brain communication, microbial metabolites have emerged as important signalling molecules. These metabolites reflect complex interactions between diet, microbial composition, and host metabolism. Two classes of metabolites, in particular, short-chain fatty acids (SCFAs) and trimethylamine N-oxide (TMAO), have attracted attention because of their potential links to neuroinflammation, vascular dysfunction, blood-brain barrier (BBB) integrity, and neurodegeneration [4-6]. These metabolites were selected for focused discussion because they are among the most extensively studied gut-derived compounds with measurable systemic levels, well-characterized metabolic pathways, and emerging human and experimental evidence linking them to neuroinflammation and vascular dysfunction.

SCFAs, primarily acetate, propionate, and butyrate, are generated through the bacterial fermentation of dietary fibres. They are known to modulate immune responses and influence epithelial and endothelial barrier function. In contrast, TMAO is produced from dietary choline and carnitine via microbial metabolism followed by hepatic oxidation. Elevated circulating TMAO levels have been associated with cardiometabolic disease and endothelial dysfunction [6]. These biological properties make both SCFAs and TMAO plausible mediators of gut-brain signalling.

Although mechanistic plausibility is strong, direct empirical evidence linking SCFAs and TMAO to cognitive outcomes remains limited and heterogeneous. Human studies are largely observational and cross-sectional, while experimental evidence is confined to animal models. Much of the existing literature consists of mechanistic and narrative reviews that synthesize conceptual frameworks and broader microbiome-Alzheimer's disease pathways and therapeutic perspectives rather than standardized empirical findings [2,3]. In this context, a focused narrative synthesis is warranted to map the emerging evidence base, integrate human and preclinical findings, and identify key gaps.

This work is presented as a narrative review rather than a systematic review; therefore, formal Preferred Reporting Items for Systematic Reviews and Meta-Analyses (PRISMA) search strategies, inclusion criteria, and risk-of-bias assessments were not applied, and evidence is synthesized qualitatively. This narrative review examines current evidence on the roles of SCFAs and TMAO in cognitive health, integrating human observational data, experimental animal studies, and mechanistic insights. The goal is not to draw causal conclusions, but to provide a balanced synthesis of what is known, what remains uncertain, and what directions future research should take.

## Review

Biology of SCFAs and TMAO

SCFAs are produced in the colon through the fermentation of non-digestible carbohydrates by anaerobic gut bacteria. Acetate, propionate, and butyrate account for the majority of circulating SCFAs. Once generated, these metabolites are absorbed into the portal circulation and exert systemic effects through multiple pathways, including the activation of G protein-coupled receptors and the modulation of histone deacetylase activity [4]. Through these mechanisms, SCFAs influence immune function, metabolic regulation, and epithelial barrier integrity.

TMAO, in contrast, originates from dietary choline, phosphatidylcholine, and carnitine. These compounds are metabolized by gut bacteria into trimethylamine, which is subsequently oxidized to TMAO in the liver by flavin-containing monooxygenases. TMAO is distributed systemically and has been implicated in atherosclerosis, endothelial dysfunction, oxidative stress, and cardiometabolic disease [6].

Gut microbiota composition plays a central role in determining both SCFA production and TMAO generation. Dysbiosis can shift microbial metabolic pathways toward reduced SCFA output or increased trimethylamine production, thereby altering host exposure to these metabolites [4,5]. These interactions underscore the importance of diet-microbiota-host crosstalk in shaping systemic metabolite profiles.

Gut microbiota, BBB integrity, and central nervous system exposure

For gut-derived metabolites to influence brain function, they must either cross the BBB or modulate peripheral pathways that affect cerebral physiology. The gut microbiota has been shown to influence blood-tissue barriers, including the BBB, through effects on endothelial tight junctions and immune signalling [1,5].

Experimental and mechanistic studies suggest that SCFAs may help preserve BBB integrity. SCFAs have been shown to modulate endothelial cell function and reduce barrier permeability in experimental models [5,7]. These effects provide a plausible pathway through which SCFAs could exert indirect neuroprotective influences.

TMAO, on the other hand, has been directly detected in human cerebrospinal fluid, demonstrating that it can access the central nervous system [8]. Further studies have shown that TMAO interacts with the blood-cerebrospinal fluid barrier, supporting biological plausibility for central exposure [9]. Together, these findings establish a mechanistic foundation for considering both SCFAs and TMAO as potential modulators of brain physiology.

Human evidence linking SCFAs to cognitive and neurological phenotypes

Direct human evidence linking SCFAs to cognitive outcomes remains limited. The most informative data come from studies in Parkinson's disease, a condition characterized by both motor and non-motor manifestations, including cognitive impairment.

Unger et al. reported that faecal SCFA levels and gut microbiota composition differed between patients with Parkinson's disease and age-matched controls. Alterations in SCFA profiles were observed alongside changes in microbial taxa, suggesting a link between microbial metabolism and neurological disease [10].

Subsequent studies extended these observations. Tan et al. demonstrated that gut microbiota composition and metabolomic profiles were associated with motor subtypes and disease severity in Parkinson's disease. Reduced abundance of SCFA-producing bacteria and altered faecal SCFA levels were linked to clinical phenotypes, including motor and non-motor features [11].

Chen et al. further reported associations between faecal and plasma SCFA levels, gut microbiota composition, and clinical severity in Parkinson's disease. Lower SCFA levels were correlated with more severe disease manifestations, reinforcing the idea that microbial metabolites may be linked to neurological vulnerability [12].

Although these findings suggest an association between SCFAs and neurological phenotypes, important limitations must be acknowledged. Most available studies remain observational and cross-sectional, precluding causal inference, and confounding factors such as diet, medication use, disease stage, renal function, and lifestyle variables complicate interpretation. However, emerging human data now extend beyond Parkinson's disease to Alzheimer's disease-related phenotypes and mild cognitive impairment, with faecal SCFA profiles associated with amyloid status, cognitive decline trajectories, and cerebrospinal fluid Alzheimer's disease biomarkers [13,14]. Despite these advances, human evidence remains limited, heterogeneous, and preliminary, and well-designed longitudinal and interventional studies are required before definitive translational conclusions can be drawn.

Human evidence linking TMAO to cognitive outcomes and neurodegeneration

Human evidence linking TMAO to cognitive outcomes has expanded over the past decade, although it remains largely observational. Del Rio et al. demonstrated that TMAO is present in human cerebrospinal fluid, providing direct evidence of central nervous system exposure [8]. Enko et al. further showed that TMAO interacts with the blood-cerebrospinal fluid barrier, supporting biological plausibility for brain effects [9].

Observational studies have linked circulating TMAO levels to neurodegenerative phenotypes. Vogt et al. reported associations between TMAO and Alzheimer's disease biomarkers, including markers of amyloid pathology and neurodegeneration [15]. Buawangpong et al. found that increased plasma TMAO levels were associated with mild cognitive impairment in an elderly population at high cardiovascular risk [16].

More recently, Yaqub et al. reported associations between TMAO and its precursors with cognition, neuroimaging features, and dementia risk. These findings suggest that TMAO-related metabolic pathways may be linked to structural and functional brain changes [17].

Despite these associations, causal inference remains limited. All of these studies are observational and subject to confounding by diet, vascular risk factors, renal function, and comorbid illness. While the consistency of associations across studies is noteworthy, human causality has not been established.

An overview of major human observational studies examining associations between gut-derived metabolites (SCFAs and TMAO) and neurological or cognitive outcomes is provided in Table 1.

**Table 1:** Human observational evidence linking SCFAs and TMAO to neurological and cognitive phenotypes SCFAs: short-chain fatty acids; TMAO: trimethylamine N-oxide; MCI: mild cognitive impairment

Author (year)	Population	Study design	Metabolite(s)	Key findings	Cognitive relevance	Major limitations
Del Rio et al. (2017) [8]	Human cerebrospinal fluid samples	Observational biochemical	TMAO	TMAO detected in human cerebrospinal fluid	Establishes central nervous system exposure	No cognitive outcomes; small sample size
Enko et al. (2020) [9]	Individuals undergoing lumbar puncture	Observational barrier study	TMAO	Interaction of TMAO with blood-cerebrospinal fluid barrier	Biological plausibility for central effects	No cognitive assessment; observational design
Unger et al. (2016) [10]	Patients with Parkinson's disease and age-matched controls	Cross-sectional observational	SCFAs	Altered faecal SCFA levels and gut microbiota composition in Parkinson's disease compared with controls	Indirect; neurological phenotype with cognitive relevance	Cross-sectional; no causality; confounding by diet, medication, and disease stage
Tan et al. (2021) [11]	Patients with Parkinson's disease	Observational cohort	SCFAs, gut metabolome	Gut microbiota and metabolomic profiles associated with motor subtypes and disease severity	Indirect; neurological phenotypes linked to cognitive vulnerability	Observational; heterogeneous phenotyping; diet and medication confounding
Chen et al. (2022) [12]	Patients with Parkinson's disease	Observational cohort	SCFAs	Faecal and plasma SCFA levels associated with gut microbiota and clinical severity	Indirect; disease severity correlates	Cross-sectional; causality unproven; metabolite measurement heterogeneity
Kuehn et al. (2025) [13]	Adults across the Alzheimer's disease continuum	Observational cohort	SCFAs (faecal)	Faecal SCFA profiles varied by amyloid status and were associated with trajectories of cognitive decline across the Alzheimer's disease continuum	Direct; linked to amyloid status and longitudinal cognitive decline	Observational design; no causal inference; potential confounding by diet, lifestyle, and comorbidities; SCFAs measured in faeces rather than plasma or cerebrospinal fluid
Nagpal et al. (2019) [14]	Subjects with MCI	Randomized crossover dietary intervention (modified Mediterranean-ketogenic diet vs. control diet)	SCFAs (faecal)	Dietary modulation of gut microbiota altered faecal SCFA profiles and was associated with changes in cerebrospinal fluid Alzheimer's disease biomarkers in subjects with MCI	Indirect; links gut-derived SCFAs to Alzheimer's disease biomarkers in MCI	Small sample size; short intervention duration; preliminary interventional evidence; no direct cognitive outcome changes demonstrated; limited generalizability
Vogt et al. (2018) [15]	Individuals with and without Alzheimer's disease	Observational cohort	TMAO	Circulating TMAO associated with Alzheimer's disease biomarkers	Direct cognitive relevance	Observational; confounding by vascular risk and renal function
Buawangpong et al. (2022) [16]	Elderly population at high cardiovascular risk	Observational cohort	TMAO	Higher plasma TMAO associated with MCI	Direct cognitive relevance	Observational; confounding by cardiometabolic disease
Yaqub et al. (2024) [17]	Community-based cohort	Observational cohort	TMAO and precursors	Associations with cognition, neuroimaging, and dementia risk	Direct cognitive relevance	Observational; residual confounding; no causal inference

Animal and preclinical evidence: TMAO

Experimental animal studies provide more direct evidence regarding the potential neurotoxic effects of TMAO. Li et al. demonstrated that TMAO supplementation promoted brain aging and cognitive impairment in mice. Treated animals showed worse memory performance and neuropathological changes [18].

Gao et al. further showed that reducing circulating TMAO levels improved cognitive function and attenuated pathological deterioration in a mouse model of Alzheimer's disease [19]. These findings suggest that TMAO may play a causal role in experimental neurodegeneration.

Deng et al. reported that higher circulating TMAO aggravated cognitive dysfunction and neuropathological changes in a rat model of vascular dementia [20]. Together, these studies provide convergent experimental evidence that elevated TMAO levels can exacerbate cognitive impairment and neuropathology in preclinical models.

However, extrapolation to humans should be cautious. Species differences in metabolism, experimental dosing, and the use of transgenic or induced disease models limit direct clinical generalizability.

Animal and preclinical evidence: SCFAs

In contrast to TMAO, SCFAs have generally shown neuroprotective effects in experimental models. Fernando et al. demonstrated that sodium butyrate reduced amyloid-β levels and improved memory performance in an Alzheimer's disease transgenic mouse model [21].

Hou et al. reported neuroprotective effects of SCFAs in a mouse model of Parkinson's disease, with improvements in motor and cognitive outcomes [22]. Liu et al. further showed that SCFAs protected against cognitive dysfunction in a model of sepsis-associated encephalopathy [23].

More recently, Zhang et al. demonstrated that engineered butyrate-producing organisms mitigated Alzheimer-like pathology and improved learning and memory in mice [24]. Together, these studies support a causal neuroprotective role for SCFAs in experimental settings.

As with TMAO, translation to human disease remains uncertain. Experimental models may not fully capture the complexity of human neurodegeneration, and dosing strategies in animals may not reflect physiologically achievable levels in humans.

The principal experimental animal studies evaluating the cognitive and neuropathological effects of SCFAs and TMAO are summarized in Table 2.

**Table 2:** Experimental animal evidence on the cognitive and neuropathological effects of SCFAs and TMAO SCFAs: short-chain fatty acids; TMAO: trimethylamine N-oxide; MPTP: 1-methyl-4-phenyl-1,2,3,6-tetrahydropyridine

Author (year)	Species/model	Intervention	Metabolite(s)	Main outcomes	Direction of effect	Translational caveats
Li et al. (2018) [18]	Mouse model of brain aging	TMAO supplementation	TMAO	Worsened memory performance; increased neuropathology	Adverse	Species differences; experimental dosing
Gao et al. (2019) [19]	Alzheimer's disease transgenic mouse model	Reduction of circulating TMAO	TMAO	Improved cognition; reduced pathological deterioration	Protective (via TMAO reduction)	Model-specific effects; translational uncertainty
Deng et al. (2022) [20]	Rat model of vascular dementia	Elevated circulating TMAO	TMAO	Aggravated cognitive dysfunction and neuropathology	Adverse	Induced disease model; species differences
Fernando et al. (2020) [21]	Alzheimer's disease transgenic mouse model	Sodium butyrate administration	SCFAs (butyrate)	Reduced amyloid-β; improved memory	Protective	Dosing relevance; transgenic model
Hou et al. (2021) [22]	MPTP-induced mouse model of Parkinson's disease	SCFA administration	SCFAs	Neuroprotection; improved neurological outcomes	Protective	Acute model; non-physiological dosing
Liu et al. (2021) [23]	Mouse model of sepsis-associated encephalopathy	SCFA supplementation	SCFAs	Protection against cognitive dysfunction	Protective	Inflammatory model; acute injury context
Zhang et al. (2025) [24]	Alzheimer's disease mouse model	Engineered butyrate-producing organisms	SCFAs (butyrate)	Improved learning and memory; reduced pathology	Protective	Novel bioengineering; early-stage evidence

Mechanistic pathways linking SCFAs and TMAO to cognitive function

Mechanistic evidence derived primarily from experimental animal models and in vitro studies provides insights into how gut-derived metabolites may influence cognitive health. SCFAs have been shown to modulate immune responses, promote regulatory T-cell differentiation, and suppress pro-inflammatory cytokine production [4,23]. Butyrate, in particular, acts as a histone deacetylase inhibitor and can influence gene expression in neural and immune cells.

SCFAs have also been implicated in the maintenance of BBB integrity and regulation of microglial maturation and activation states [4,7]. Through these mechanisms, SCFAs may exert broadly neuroprotective effects by dampening neuroinflammation and preserving neuronal homeostasis.

In contrast, TMAO has been linked to endothelial dysfunction, oxidative stress, and activation of inflammatory signalling pathways [6]. These effects may compromise cerebral perfusion and exacerbate neurovascular dysfunction. Observational human studies and experimental animal data further implicate TMAO in pathways relevant to neurodegeneration [15,18].

Together, these mechanistic pathways provide a coherent biological framework that aligns with the direction of associations observed in human and animal studies. However, most mechanistic insights remain conceptual and require validation in well-designed human experiments.

The major mechanistic pathways linking gut microbiota-derived metabolites to cognitive function are synthesized in Table 3.

**Table 3:** Mechanistic pathways linking gut microbiota-derived metabolites to cognitive function SCFAs: short-chain fatty acids; TMAO: trimethylamine N-oxide

Pathway	Metabolite(s)	Proposed effect	Evidence type	Key references	Evidence strength
Immune modulation	SCFAs	Suppression of pro-inflammatory cytokines; promotion of regulatory T cells	Experimental + mechanistic reviews	Silva et al. (2020) [4]; Liu et al. (2021) [23]	Moderate
Microglial regulation	SCFAs	Regulation of microglial maturation and activation	Mechanistic review	Silva et al. (2020) [4]	Moderate
Blood-brain barrier integrity	SCFAs	Preservation of endothelial tight junctions; reduced permeability	Experimental + mechanistic reviews	Tang et al. (2020) [5]; Fock and Parnova (2023) [7]	Moderate
Endothelial dysfunction	TMAO	Promotion of oxidative stress and endothelial injury	Mechanistic review + experimental	Janeiro et al. (2018) [6]; Li et al. (2018) [18]	Moderate
Neurovascular impairment	TMAO	Compromised cerebral perfusion; vascular contributions to neurodegeneration	Observational + mechanistic	Tang et al. (2020) [5]; Vogt et al. (2018) [15]	Low-moderate
Central nervous system exposure	TMAO	Penetration of blood-cerebrospinal fluid barrier	Human observational	Del Rio et al. (2017) [8]; Enko et al. (2020) [9]	High (for exposure only)

Secondary synthesis and meta-analytic context

Higher-level summaries provide additional context regarding the relationship between TMAO and cognitive outcomes. Long et al. conducted a systematic review and meta-analysis examining associations between TMAO and cognitive impairment [25]. Ren and Mo similarly reported associations between circulating TMAO levels and cognitive dysfunction [26].

These secondary syntheses suggest that associations between TMAO and cognitive impairment are reproducible across populations. However, both reviews emphasized substantial heterogeneity in study design, metabolite measurement methods, and cognitive outcome measures. Importantly, the evidence remains observational and does not establish causality.

Translational implications

Although much of the current support comes from experimental animal and preclinical studies, the emerging evidence raises the possibility that SCFAs and TMAO could serve as candidate biomarkers or therapeutic targets in cognitive disorders. SCFAs, particularly butyrate, may represent neuroprotective metabolites with potential relevance for disease modification [4,21,22].

Conversely, TMAO may represent a risk-associated metabolite. Observational human studies and experimental animal data consistently link higher TMAO levels to adverse neurological and cognitive outcomes [15,16,18].

Dietary modulation of fibre intake could influence SCFA production, while strategies to reduce TMAO levels have been proposed in cardiometabolic disease contexts [6]. Despite these theoretical possibilities, clinical translation remains premature. The lack of robust human intervention data and the observational nature of existing evidence preclude clinical recommendations at this stage.

Limitations of the current evidence

Several limitations constrain the current understanding of the roles of SCFAs and TMAO in cognitive health. Human evidence remains sparse and largely observational, although emerging studies now link SCFAs to Alzheimer's disease-related phenotypes and mild cognitive impairment; nonetheless, direct causal evidence remains lacking.

Much of the available literature consists of animal and mechanistic studies, which may not fully capture the complexity of human neurodegenerative disease. There is substantial heterogeneity in methods used to quantify SCFAs and TMAO, as well as in cognitive outcome measures across studies.

Finally, the absence of well-designed longitudinal studies and human intervention trials limits causal inference and clinical translation.

Future directions

Future research should prioritize longitudinal cohort studies that simultaneously assess gut microbiota composition, circulating and faecal metabolite levels, and detailed cognitive phenotypes. Standardized metabolomics platforms and harmonized cognitive assessments would improve comparability across studies [12,23].

Small interventional studies in mild cognitive impairment suggest that dietary modulation of gut microbiota and SCFA production may influence Alzheimer's disease biomarkers, underscoring the need for larger, adequately powered randomized trials in prodromal neurodegenerative populations [14].

Human interventional trials targeting dietary fibre intake, probiotic supplementation, or microbial enzyme pathways may help establish causal relationships between gut-derived metabolites and cognitive outcomes [4,6]. Mechanistic validation in human tissues and imaging studies could further clarify how SCFAs and TMAO influence neuroinflammatory and neurovascular processes.

## Conclusions

Evidence linking gut microbiota-derived metabolites to cognitive health is emerging but remains preliminary. SCFAs appear broadly neuroprotective in experimental models, whereas TMAO shows adverse associations, particularly in preclinical studies.

Human evidence remains limited and observational, and causality has not been established. At present, clinical translation is premature. SCFAs and TMAO should be regarded as promising but unproven candidates in the complex landscape of gut-brain interactions. Well-designed longitudinal and interventional studies are required before these metabolites can be considered reliable biomarkers or therapeutic targets.
